# The Brilliance of the Zebrafish Model: Perception on Behavior and Alzheimer’s Disease

**DOI:** 10.3389/fnbeh.2022.861155

**Published:** 2022-06-13

**Authors:** Avinash Shenoy, Meheli Banerjee, Archana Upadhya, Siddhi Bagwe-Parab, Ginpreet Kaur

**Affiliations:** Shobhaben Pratapbhai Patel School of Pharmacy & Technology Management, SVKM’s Narsee Monjee Institute of Management Studies, Mumbai, India

**Keywords:** zebrafish, Alzheimer’s disease, behavior, glutamatergic, cholinergic

## Abstract

Alzheimer’s disease (AD) has become increasingly prevalent in the elderly population across the world. It’s pathophysiological markers such as overproduction along with the accumulation of amyloid beta (Aβ) plaques and neurofibrillary tangles (NFT) are posing a serious challenge to novel drug development processes. A model which simulates the human neurodegenerative mechanism will be beneficial for rapid screening of potential drug candidates. Due to the comparable neurological network with humans, zebrafish has emerged as a promising AD model. This model has been thoroughly validated through research in aspects of neuronal pathways analogous to the human brain. The cholinergic, glutamatergic, and GABAergic pathways, which play a role in the manifested behavior of the zebrafish, are well defined. There are several behavioral models in both adult zebrafish and larvae to establish various aspects of cognitive impairment including spatial memory, associative memory, anxiety, and other such features that are manifested in AD. The zebrafish model eliminates the shortcomings of previously recognized mammalian models, in terms of expense, extensive assessment durations, and the complexity of imaging the brain to test the efficacy of therapeutic interventions. This review highlights the various models that analyze the changes in the normal behavioral patterns of the zebrafish when exposed to AD inducing agents. The mechanistic pathway adopted by drugs and novel therapeutic strategies can be explored *via* these behavioral models and their efficacy to slow the progression of AD can be evaluated.

## Introduction

Globally there has been a rise in the occurrence of neurological disorders such as Alzheimer’s disease (AD), an advancing multifaceted neurodegenerative disorder, is the leading cause for 70% of dementia worldwide (Mayeux and Stern, 2012). The number of patients suffering from AD is predicted to reach 78 million globally by 2030 and expected rise to 139 million by 2050 (World Health Organization, 2021). The two main pathophysiological characteristic features of AD are formation of amyloid beta (Aβ) plaques and intracellular neurofibrillary tangles (NFT) (Sajjad et al., 2018; Hampel et al., 2021). Extracellular deposits of amyloid plaques form in the brain parenchyma and cerebral blood vessels, a condition termed as congophilic angiopathy or cerebral amyloid angiopathy (CAA)(Thanvi and Robinson, 2006; Jäkel et al., 2021). NFT are intracellular, large paired helical filaments of hyperphosphorylated tau proteins which cause synaptic and neural loss (Moloney et al., 2021). The predominant regions of the human brain which display AD pathology are the association areas of the cerebral cortex and the hippocampus (Wang et al., 2010). The vulnerable neurons among these regions are in the layer II of the entorhinal cortex, the subiculum and the CA1 region of the hippocampus since they are susceptible to display high levels of NFT and are first to be lost in early phases of the disease (Hyman et al., 1984; Kordower et al., 2001; Price et al., 2001). AD associated neuronal loss is observed, at specific cortical and subcortical brain sites such as the *trans-*entorhinal and entorhinal regions, hippocampus, amygdala, medial septal nucleus, nuclei of the diagonal band of Broca, basal nucleus of Meynert, compact part of the substantia nigra, locus coeruleus, midbrain, and pontine raphe nuclei. The neuronal loss is predominantly due to intra-neuronal deposits and extra-neuronal deposits of abnormal protein, which constitute the irregularly phosphorylated tau protein and the insoluble beta amyloid protein, respectively (Arnold et al., 1991; Stratmann et al., 2016). Further, dysregulation of neurochemicals such as acetylcholine (Ach), dopamine, glutamate, gamma amino butyric acid (GABA), serotonin and noradrenaline, due to neuronal loss in these critical regions has been observed in AD (Kaur et al., 2019). The manifested behavior in AD patients depend on the nature of development of the disease and progressively includes memory lapses, difficulty in organizing and planning of tasks, confusion, disorientation, changes in sleep patterns, anxiety, hallucinations, delusions, paranoia and lack of physical control (Mega et al., 1996; Serda, 2013). The neuropsychiatric symptoms (NPS) are based on disruptions in frontal-subcortical circuits (involving frontal cortex, basal ganglia, thalamus), cortico-cortical network (with hippocampus and amygdala as the epicenters), and the monoaminergic system [comprising of the neuronal cell bodies producing serotonin, norepinephrine, or dopamine located primarily in the brain stem (midbrain, pons, and medulla) and diffusely projected *via* long axons to virtually all parts of the brain to mediate human behavior] (Geda et al., 2013). These three neurobiological models form the foundations of neuroimaging and biomarker research in AD. It can be characterized into two main types, familial AD (FAD) and sporadic AD (SAD) (Piaceri et al., 2013).

Previously, AD animal models were developed to reproduce the brain lesions formed due to Aβ plaques and NFTs with the assumption that the models would authentically reproduce the human clinical condition. However, many therapeutics which have shown promise in the animal models have failed to show efficacy in clinical trials (Franco and Cedazo-Minguez, 2014). The challenges to translatability of the experiments include lack of good predictable animal models, lack of good biomarkers to indicate disease progression and diversity of genetic susceptibility of the target population under clinical trials (Franco and Cedazo-Minguez, 2014). The drawbacks of AD animal models such as rodents (including transgenics), canines, non-human primates for translation research are well discussed by Vitek and colleagues (Vitek et al., 2020) and will not be reiterated here. Emphasis, is on the development of current AD models that accelerate the translatability of the therapeutic interventions to check the progression of the disease (Götz et al., 2018; Li et al., 2019).

Zebrafish (*Danio rerio)* which belongs to the infraclass of teleost fishes is found to have certain features that makes it a suitable model for the study of AD and evaluation of therapeutic agents. Zebrafish possesses the following features; genes orthologous to the genes known to be involved in AD (Newman et al., 2011), a neuroanatomic alignment like humans (Guo, 2009; Kozol et al., 2016; Gupta et al., 2018) comparable neural signaling (Horzmann and Freeman, 2016) and the propensity of adult zebrafish and larval zebrafish to serve as models to study behavior (Norton and Bally-Cuif, 2010; Fero et al., 2011). These similarities make zebrafish an exceptional model for studying various neurodegenerative diseases comparable to *in vivo* and *in vitro* mammalian models for therapeutic drug screening (Michael Stewart and Kalueff, 2012). In recent years, the zebrafish model has proved to be useful in the study of neurodegeneration due to AD (Thawkar and Kaur, 2021). This review highlights the behavioral model development in both adults and larval zebrafish which enable the comprehension of AD progression mechanisms and efficacy of therapeutic interventions.

## Zebrafish as an Alzheimer’s Disease Model

Zebrafish has several characteristics that make it a suitable model for drug discovery. The zebrafish female can produce 200–250 eggs per mating, the embryogenesis is rapid i.e., the entire body structure is made 24 h post fertilization (hpf) and the internal organs like heart, liver and kidney develop 96 hpf. The development of the zebrafish can be visualized *in vivo* due to the transparency of the larvae. These fishes can be genetically manipulated and the gene expressions can be studied using adequate photo-imaging tools. Further, zebrafish embryos can be used to screen potential compounds in 50 microliter (μl) volumes (Chakraborty et al., 2009) (similar to an *in vitro* cellular model). Compounds added to water containing zebrafish, undergo the processes of absorption, distribution, metabolism and excretion similar to an *in vivo* model (Bhusnure et al., 2015). Zebrafish being a vertebrate, is evolutionary closer to humans than that of drosophila or the nematode models which are popular in neuroscientific and genetic studies (Shams et al., 2018). Using zebrafish in research, in lieu of animal studies involving mammals, is advantageous since it fulfils the principle of 3R’s; replacement, refinement and reduction. The European Commission Directive in 2010, exempts the studies with larval zebrafish up to 5 days post fertilization (dpf) from regulatory protection (Cassar et al., 2020). Therefore, studies with zebrafish larvae up to 5 dpf can be carried out as an alternative to testing in higher animals.

Thus, zebrafish applications in neurodegenerative disease research and neuropharmacology are greatly expanding due to lower economic costs, the small size of the organism, a sequenced characterized genome, and well described anatomical structures (Basnet et al., 2019). The most advantageous characteristics of zebrafish as a model would be the optical clarity of the embryo which would allow phenotypic visualization of the effects of genes all through the developmental process and the ease of introducing transient genetic manipulations and chemical manipulations (Koster and Sassen, 2015). All of these characteristics coupled with the presence of several orthologous genes in humans make zebrafish an ideal model to study AD as compared to rodents (Saleem and Kannan, 2018).

### Neuroanatomical Comparisons Between Zebrafish and Mammalian Brain

**FIGURE 1:**
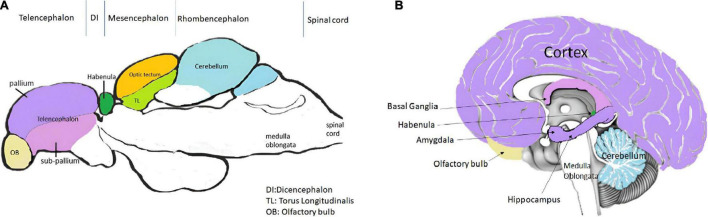
Comparison of panel **(A)** (a representative of the mid-sagittal section of zebrafish brain) with **(B)** (a representative of mid-sagittal section of human brain). The figure attempts to represent the important areas involved in learning and memory in humans and zebrafish (Bally-Cuif and Vernier, 2010; Kozol et al., 2016; Langova et al., 2020).

Cognition includes all the mental processing by which knowledge is acquired, retained and used in perception, learning, memory and thinking. Despite not having a defined cortex or hippocampus like the mammalian brain, zebrafishes can perform required learning and memory tasks *via* various parts in brain that are functionally equivalent to these structures (Calvo and Schluessel, 2021; Figure 1 and Table 1.

**TABLE 1:** Comparison of regions of mammalian brains with their homologous counterpart in zebrafish.

Sr. No	Mammalian brain	Homologous regions in zebrafish brain	Function in zebrafish	References
1.	Iso-cortex and the transitional cortex	Dorsal pallium	Control of short- term memory processes	Vargas et al., 2009
2.	Basal ganglia	Sub-pallium	Cognitive functions essential adaptive behavior such as planning, attention, learning and behavior	Cheng et al., 2014; Medina et al., 2014
3.	Hippocampus and amygdala	Lateral pallium and medial pallium	Control of sensory, motor and cognitive functions, like memory, learning and emotion	Ganz et al., 2015
4.	Habenula	Habenula	Control of motor and cognitive behaviors	Amo et al., 2010; Bühler and Carl, 2021
5.	Superior colliculus	Optic tectum	Control of vision	Wullimann et al., 1996
6.	Inferior colliculus	Torus longitudinalis	Control of hearing	Wullimann et al., 1996
7.	Medulla oblongata	Medulla oblongata	Control of respiration, circulation and wakefulness	Moens and Prince, 2002
8.	Cerebellum	Cerebellum	Control of motor reflexes, emotional learning and spatial cognition	Rodríguez et al., 2021

From a structural perspective, the zebrafish brain is similarly aligned as the mammalian brain possessing a forebrain (anterior most part), midbrain, hind brain (posterior most part) and a spinal cord (Guo, 2009). These develop at 24 hpf in the zebrafish. The forebrain of the zebrafish embryo further forms the telencephalon (composed by the pallium, the sub-pallium and the olfactory bulbs), the diencephalon, the hypothalamus and the retina (Vaz et al., 2019). The sub-pallium is the ventral telencephalon while the pallium is the dorsal telencephalon. The ventral telencephalon is sub-divided into two brain nuclei; the ventral nucleus (Vv) and the dorsal nucleus (Vd) of the ventral telencephalon. The dorsal telencephalon is much more complex and is composed of different brain nuclei or regions; the central zone (Dc), the dorsomedial zone (Dm), the lateral zone (Dl) and the posterior zone (Dp) of the dorsal telencephalon (Wullimann et al., 1996; Diotel et al., 2020). The zebrafish brain develops by eversion rather than inversion, due to which some classical regions of the mammalian brain, such as the hippocampus, amygdala, and substantia nigra are not present but the fish brain has structures which can carry out similar functions (Schmidt et al., 2013). Among the estimated 16 neurogenic niches of the zebrafish telencephalon, the Vv of the subpallium is comparable to the sub-ventricular zone (SVZ) of the lateral ventricle of mammals and the Dl, Dp are thought to be equivalent to sub-granular zone (SGZ) between the dentate gyrus (DG) and hilum of the mammalian hippocampus (Diotel et al., 2020; Ghaddar et al., 2021). The lateral pallium (LP) is predicted to be important for spatial learning, whereas the medial pallium (MP) is integral for avoidance learning in the zebrafish (Calvo and Schluessel, 2021). The diencephalon comprises of the thalamus, pineal body, and habenula (Vaz et al., 2019). The forebrain is responsible for receiving and processing sensory information and directing behavior. Telencephalon regulates social behavior, memory and emotion (Broglio et al., 2011) while the diencephalon addresses attention, alertness and circadian actions. Though the structures of telencephalon and diencephalon of the teleost brain are not seen in mammalian brain, some regions of the zebrafish and mammalian forebrain are conserved with respect to architecture and function (Vaz et al., 2019).

The midbrain (mesencephalon) of the zebrafish is important for vision and hearing (Vaz et al., 2019). It lies between the forebrain and hindbrain, containing the optic tectum, torus semicircularis, torus longitudinalis and the midbrain tegmentum (Zebrafish UCL, 2022). The region in mammalian brain functionally similar to the optic tectum, which has a role in vision in zebrafish, is known as the superior colliculus. The inferior colliculus of mammalian brain can be considered to be comparable to the torus longitudinalis which is responsible for auditory sensations in the zebrafish (Wullimann et al., 1996).

The hindbrain (rhombencephalon) of the zebrafish is composed of the posteriorly located medulla oblongata, the ventro-anterior pons and the dorso-anterior cerebellum (Moens and Prince, 2002). The cerebellum of the zebrafish is sub-divided into three parts; the vestibulolateralis lobe, the corpus cerebelli and the valvula cerebelli (Wullimann et al., 1996). The cerebellum controls motor reflexes and plays a role in emotional learning and spatial cognition in teleost fishes (Rodríguez et al., 2005). The fourth ventricle of the brain is formed by the medulla and pons which clubbed with the mid brain are referred to as the “brain stem.” The brain stem regulates respiratory, circulatory and wakefulness activities in the teleost (Moens and Prince, 2002).

Functionally analogous regions in the brains of zebrafish and humans are illustrated and outlined in Figure 1 (Bally-Cuif and Vernier, 2010; Kozol et al., 2016; Langova et al., 2020).

### Neurotransmitter Pathways in Mammals and the Zebrafish

AD in humans is caused by excessive neuronal loss predominantly in the hippocampus and the cerebral cortex resulting into cognitive dysfunction, memory loss and behavioral changes in the patient. The symptoms of this disease are exacerbated when there is a massive loss of cholinergic neurons that synthesize Ach, a neurotransmitter involved in memory consolidation (Kaur et al., 2019). The currently approved therapy for AD includes acetylcholinesterase inhibitors (rivastigmine, galantamine, and donepezil), and a N-methyl D-aspartate receptor antagonist (NMDA) (e.g. memantine), and these are thought to preserve cholinergic neurotransmission (Guzior et al., 2014; Kaur et al., 2019) Besides ACh (Tohgi et al., 1994) several other neurotransmitters such as gamma amino butyric acid (GABA) (Zimmer et al., 1984), glutamate (Kuiper et al., 2000), serotonin (Volicer, 1985), dopamine (Pinessi et al., 1987) are present in reduced levels in the cerebrospinal fluid (Kaur et al., 2019) of AD patients and may contribute to the pathology of the disease.

Some AD patients exhibit extrapyramidal symptoms suggesting a loss of dopaminergic neurons (Lopez et al., 1997; Martorana and Koch, 2014). Aβ plaques, NFT, neuronal loss, and a reduction in dopamine content were observed in the neurons constituting the nigrostriatal Pathway. Thus, indicating that dopamine is clearly involved in the pathogenesis of AD. The zebrafish central nervous system uses similar neurotransmitters as the mammalian brain (GABA, glutamate, dopamine, noradrenaline, serotonin, histamine, and ACh) in both interneuron systems and in nerve pathways (Panula et al., 2006, 2010).

The differences and similarities in the metabolic and synthetic routes of these neurotransmitters in zebrafish and mammals are well reviewed by Horzmann and Freeman (2016) and Wasel and Freeman (2020). Three well researched neurotransmitter pathways i.e., cholinergic, glutamatergic, and GABAergic implicated in AD in zebrafish and humans (Santana et al., 2012) are described briefly.

#### Cholinergic Pathway

Ach is a significant neurotransmitter that is quite widespread in the human central nervous system (CNS) (Bierer et al., 1995). It is essential in learning processes and functions of memory and also helps in regulating the release of other neurotransmitters such as GABA. AD symptoms are manifested after a loss in cholinergic transmission in hippocampal region and the basal areas of the forebrain (Auld et al., 2002; Geula et al., 2021). The presence of acetylcholinesterase neurons in the telencephalon suggests the prevalence of a cholinergic system in the teleost. The telencephalon is a key area in the zebrafish brain for memory consolidation (Flood et al., 1976). The presence of cholinergic neurotransmission pathways has been validated in zebrafish by previous research as well (Williams and Messer, 2004; Mans et al., 2019). Studies using electrophysiological methods, histological methods, and antibody binding methods have indicated that the cholinergic system is widely distributed in the zebrafish brain (Mueller et al., 2004).

#### Glutamatergic Pathway

Glutamatergic pathway involving the NMDA receptor is one of the important excitatory pathways in vertebrates and is essential in learning, memory, and synaptic plasticity (Li and Tsien, 2009). However, increased amounts of glutamate in synaptic cleft can be neurotoxic (Maragakis and Rothstein, 2004).

Glutamate is a ligand to some classes of metabotropic receptors and ionotropic receptors. Human ionotropic glutamate receptors (iGluRs) include, NMDA, amino-3-hydroxy-5-methyl-4-isoxazolepropionic acid (AMPA), and kainate receptors (Hu et al., 2012). Among ionotropic receptors, the NMDA receptor has been well characterized in zebrafish. Studies further proved that the telencephalon is sensitive to antagonists of NMDA receptors and that its long-term stimulation affects both memory and learning skills (Nam et al., 2004).

Reports on a family of excitatory amino acid transporters (EAATs) in humans, which transport the glutamate away from the synapse, have indicated that, neutralization of excess glutamate could reduce the excitotoxicity of the neuroreceptor (Danbolt, 2001). The presence of EAAT-orthologous genes in zebrafish makes it an ideal model to study this pathway (Rico et al., 2010).

#### GABAergic Pathway

GABA is an inhibitory neurotransmitter which regulates neural functions *via* modulating the activity of postsynaptic cells (Bowery and Smart, 2006). Defects in GABA neurotransmission plays a major role in CNS disorders (Staley et al., 1995). The GABAergic system is extensively present throughout the brain and plays a major role in balancing excitatory signals with inhibitory signals (Rissman et al., 2007). The equilibrium between the two is important in synchronization of various CNS functions. Through various pre-clinical and clinical studies it has been established that dysfunction of GABAergic system causes imbalance in excitatory and inhibitory signals. This imbalance is one of the potential markers for the initial stages of AD (Zheng et al., 2021). Neurodegenerative diseases can result from dysfunctional brain cells leading to changes in signaling systems (Lanctôt et al., 2004). The presence of GABAergic neurons and GABA_A_ and GABA_C_ receptors in zebrafish has been reported in the telencephalon, hypothalamus, tectum striatum, and olfactory bulb (Sadamitsu et al., 2021). Glutamic acid decarboxylase (GAD) is an enzyme that is conserved over species (Bosma et al., 1999). Reports of genes similar to human GAD genes in zebrafish were recorded and also their early stage expression was observed. GAD enzyme was majorly concentrated in the areas of the telencephalon medial longitudinal fasciculus in the midbrain, and at the border regions of the rhombomeres in the rostral hindbrain (Martin et al., 1998).

**FIGURE 2:**
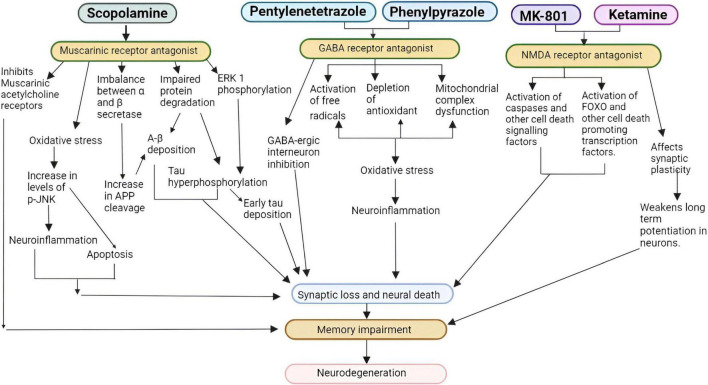
Pathways for cholinergic, glutamatergic, and GABAergic inducers (FOXO, forkhead box transcription factor, p-JNK, p-Jun N-terminal kinase, APP, amyloid precursor protein, NMDA, N-methyl-D- aspartate, GABA, γ-amino butyric acid ERK1, Extracellular signal-regulated kinase 1) (Guan, 2008; Chen and Yeong, 2020).

Administration of drugs and chemicals can modify the neurotransmission pathways by targeting various molecular mechanisms. Several compounds induce AD in mammals *via* one of these pathways (Cholinergic, Glutamatergic, and GABAergic). Figure 2 elaborates the possible pathways by which certain inducers dysregulate signaling to promote AD.

## Genes Implicated in Human and Zebrafish Alzheimer’s Disease

Multiple studies have discovered that in FAD autosomal dominant mutations are mainly in three genes that are presenilin 1 (PS1), presenilin 2 (PS2), and Aβ precursor protein (APP) (Soto-Ospina et al., 2021). The expressed proteins of these genes are involved in ensuring functional cleavage of APP genes or in the formation of soluble Aβ protein. When mutated these proteins lead to the formation of insoluble Aβ which leads to Aβ plaque accumulation (O’Brien and Wong, 2011). SAD is correlated with the expression of the apolipoprotein E (ApoE) variant, ε4 (Xia, 2010). In zebrafish, various homologous gene encoding such as PS1, PS2, APP, and ApoE have been identified (Newman et al., 2014; Kiper and Freeman, 2021). Table 2 enlists the genes that have a prominent role in AD and their orthologs in zebrafish.

**TABLE 2:** Orthologs of major human genes implicated in AD in the zebrafish.

Genes in humans	Function of gene	Gene Orthologs or co-orthologs in zebrafish	#Percentage identity of the expressed protein	References
*PSEN1* *(Presenilin 1)* (Uniprot ID: P49768)	Catalytic subunit of gamma secretase complex that aids in the cleavage of APP	*psen1* (Uniprot ID: Q9W6T7)	72.4	Leimer et al., 1999; Fraering et al., 2004
*PSEN2* *(Presenilin 2)* (Uniprot ID: P49810)	Catalytic subunit of gamma secretase complex that aids in the cleavage of APP	*psen2* (Uniprot ID: Q90ZE4)	71.3	Groth et al., 2002; Tu et al., 2006
*APP* *(Amyloid Precursor Protein)* (Uniprot ID: P05067)	Cell surface receptor that aids in neurite growth, neuronal adhesion and axonogenesis. The interaction of APP molecules on nearby cells promote synapse formation	*appa* (Uniprot ID: Q90W28)	72.65	Musa et al., 2001; Baumkotter et al., 2014
		*appb* (Uniprot ID: B0V0E5)	68.44	
*MAPT* *(Microtubule associated protein tau)* (Uniprot ID: P10636)	Promotes the stability and assembly of microtubules which help in establishing and maintaining neuronal polarity. It acts as a linker between C-terminal (binds axonal microtubules) and N-terminal (binds the plasma membrane components of the neuronal cells) of microtubules.	*mapta* *(Uniprot ID: Not assigned)*		Chen et al., 2009; Yoshida and Goedert, 2012; Sandberg et al., 2020
		*maptb* *(Uniprot ID: Not assigned)*		
*APOE* *(Apolipoprotein E)* (Uniprot ID: P02649)	Plays a role in lipid homeostasis. It regulates lipid transport in the CNS which aids in neuron survival and sprouting.	*apoea* (Uniprot ID: Q503V2)	21.57	Fagan et al., 1996; Babin et al., 1997; Kowal et al., 1989; Sehayek and Eisenberg, 1991; Huang and Mahley, 2014; Kockx et al., 2018
		*apoeb* (Uniprot ID: O42364)	20.30	
*(PSENEN)* *(Presenilin Enhancer, Gamma-Secretase Subunit)* (Uniprot ID: Q9NZ42)	Essential subunit of gamma secretase complex that aids in the cleavage of APP	*psenen* (Uniprot ID: Q8JHF0)	77	Edbauer et al., 2003; Kimberly et al., 2003; Campbell et al., 2006; Zhou et al., 2019
*BACE1* *(Beta Secretase 1)* (Uniprot ID: P56817)	Proteolytic cleavage of APP at the N-terminal between 671th and 672th residue which leads to the generation of beta cleaved soluble APP	*bace1* (Uniprot ID: A0A0G2KH37)	75.3	Hussain et al., 1999; Lin et al., 2000; Okada et al., 2010; Moussavi Nik et al., 2012
*BACE2* *(Beta Secretase 2)* (Uniprot ID: Q9Y5Z0)	Proteolytic cleavage of APP between residues 690 and 691 also 671 and 672	*bace2* (Uniprot ID: Q5XJ89)	59.84	Esterházy et al., 2011; van Bebber et al., 2013
*NCSTN* *(Nicrastin)* (Uniprot ID: Q92542)	Essential subunit of gamma secretase complex that aids in the cleavage of APP	*Ncstn* (Uniprot ID: B3DGT7)	56.36	Yu et al., 2000; Edbauer et al., 2003; Lu et al., 2014; Bai et al., 2015; Lim et al., 2015; Zhou et al., 2019

*^#^Multiple alignment done by BLAST (https://blast.ncbi.nlm.nih.gov/Blast.cgi) and values obtained from the alignment scores.*

## Behavioral Models of Alzheimer’s Disease in Zebrafish

**FIGURE 3:**
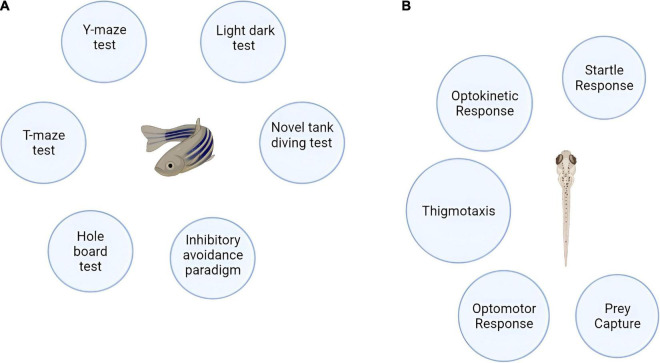
**(A)** Behavioral models of adult zebrafish. **(B)** Behavioral models of zebrafish larvae.

Symptoms of AD in humans span from cognitive deficits (evidenced by decrease in memory, spatial recognition, problem solving, and language), abnormal motor movements (tremors, loss of co-ordination, incontinence, eating troubles) to behavioral and emotional issues (depression, agitation, anxiety, depression, tendency to hallucinate) (Voisin and Vellas, 2009; Blanc et al., 2014). The zebrafish when exposed to an AD inducing drug also manifest cognitive and memory impairments. The behavioral responses in the zebrafish can be grouped into basic motor responses which is inclusive of observed sensorimotor responses which in turn is encompassed by the higher learning and memory related reactions of the fish. The types of fish responses and the observed endpoints for behavioral assessments are well described in the publication by Tierney (2011). Major behavioral endpoints are conducted on the zebrafish 3–4 days post fertilization except learning or memory dependent endpoints (Tierney, 2011). The behavioral tests that can be performed on the zebrafish are shown in Figure 3. The AD inducing agents, effects of the moieties counter-acting AD in zebrafish and the behavioral model used for assessment are outlined in Table 3.

**TABLE 3:** Table for zebrafish pathways, models, and drugs that affect those pathways.

AD Inducing agents	Age of zebrafish	Drugs against AD inducing agent	Molecular mechanism of the anti-AD drug	Behavioral tests conducted	References
Scopolamine	Adult 6 months	Li_2_CO_3_ (100 mg/L, 7 days)	Decrease p tau	Novel tank and Y-maze	Zanandrea et al., 2018
	Adult 3–4 months	*Thymus vulgaris* L essential oil (25–300 uL/L, 14 days)	Reduction in AChE activity and brain antioxidant capacity	Novel tank test, novel object recognition and Y-maze	Capatina et al., 2020
	Adult 6–8 months	Cotinine/6- hydroxy- L-nicotine (1–2 mg/L, 10 days)	Reduces oxidative stress and AChE activity and upregulates neuroprotective genes.	Novel tank diving, Y-maze and Object discrimination	Boiangiu et al., 2021
	Adult	Physostigmine (20 uM, 48 h)	AChE inhibitor and anxiolytic effect.	Passive avoidance	Kim et al., 2010
	Adult	*Streblus asper* 200–800 mg/kg 7 days	AChE inhibitor	Color-Biased Appetite Conditioning T-Maze and inhibitory avoidance	Singsai et al., 2021
	Larvae 168 hpf	*Convolvulus pluricaulis* 0.38 mg/ml 1 h	AChE inhibitor		Karunakaran et al., 2022
	Larvae 3dpf	Apigenin-rivastigmine hybrids 12.5 μg/mL	Antioxidant property, inhibits Aβ aggregation and exhibits anti-inflammatory property	Y-maze	Sang et al., 2020
	Adult 3-4 months	Hydroethanolic Extract of *Lycopodium selago* L. 3 mg/L 8 days	Inhibits AChE and has antioxidant properties	Y-maze, Novel tank and Novel object recognition	Valu et al., 2021a
	Adult 6–8 months	*Glycyrrhiza glabra* extract (250 mg/L) 30 mins	Cognitive improvement	T-maze and Novel object preference	Pusceddu et al., 2022
	Adult = 6 months	*Hericium erinaceus* ethanolic extract 3 mg/L 13 days	Enhances nerve growth factor (NGF) mRNA, increases protein expression in hippocampus along with antioxidant properties.	Y-maze, novel tank diving and novel object recognition	Valu et al., 2021b
	Adult < 8 months	Quercetin 20 ml/kg Single dose after 1 h exposure to scopolamine	Antioxidative property *via* free radical scavenging	Inhibitory avoidance and exploratory assessment	Richetti et al., 2011
	Adult <8 months	Rutin 20 ml/kg Single dose after 1 h exposure to scopolamine	Antioxidative property via free radical scavenging	Inhibitory avoidance and exploratory assessment	Richetti et al., 2011
	Adult 3–4 months	Agathisflavone 1–5 μg/L 8 days	Inhibits AChE activity and displays antioxidant activity	Novel tank diving and Y-maze	Dumitru et al., 2019
Aluminum chloride	Larvae 3 dpf	Linarin 16.7 μg/mL 3–5 dpf	Inhibits AChE activity	–	Pan et al., 2019
	Larvae 72 hpf	3-[4-(4-chloromethyl-benzoylamino)-phenyl]-8-methoxycoumarin 50 μg/mL 3 days	Inhibits AChE activity	Locomotor activity	Hu et al., 2019
	Larvae 2 dpf	Compound 4e 0.8 μg/mL 2–5 dpf	Inhibits Aβ_1–42_ aggregation	–	Wang et al., 2021
	Larvae 2 dpf	TM-10 (Ferulic acid derivative) 0.33 μg/mL Single dose at 3 dpf	Inhibits Aβ_1–42_ aggregation	–	Sang et al., 2019
	Larvae 3 dpf	*Cistanche tubulosa* (Schenk) wight 30 μg/ml 5 days	Inhibition of neuroinflammation by targeting TNF-α and IL-1β	–	Li et al., 2020
	Adult 6–8 months	Necrostatin-1 15 μmoL/L 30 days	Blocks necroptotic cell death by inhibiting receptor-interacting protein kinase-1 (RIP-1)	T-maze	Gao et al., 2022
	Adult 1 year old	Plasmalogen 3 mg/fish/day 8 weeks	Alleviating oxidative stress	Locomotor activty	Feng et al., 2021
	Juvenile 72 hpf	3-arylcoumarin Compound 2 (100 μg/ml) Compound 20 (50 μg/ml) Compound 22 (100 μg/ml)	Inhibition of monoamine oxidase B	Locomotor activity	Yang et al., 2019
	Larvae 3 dpf	Apigenin-rivastigmine hybrids 12.5 μg/mL	Antioxidant property, inhibits Aβ aggregation and exhibits anti-inflammatory property	Y-maze	Sang et al., 2020
Okadaic acid	Adult 12-15 months	4-benzyl-2-methyl-1, 2, 4-thiadiazolidine-3, 5- dione 1μM 9 days	Normalizes PP2A activity, phosphorylated tau and inhibition of GSK3β	Learning and memory	Koehler et al., 2019
	Adult 12-15 months	Lanthionine ketimine-5-ethyl ester 500 μM 9 days	Increasing levels of Brain-derived neurotrophic factor, Protein kinase B and cAMP response element-binding protein. Decreases apoptosis.	Learning and memory	Koehler et al., 2018
Aβ injection	Larvae 1 dpf	LiCl 100 μM 1–5 dpf	Decrease of p-tau	Locomotor and bouncing ball avoidance	Nery et al., 2014
	Larvae 2 dpf	TM-10 (Ferulic acid derivative) 0.33 μg/mL Single dose at 3 dpf	Inhibits butyrylcholinesterase activity, monoamine oxidase activity and aggregation of Aβ	–	Sang et al., 2019
	Larvae 5 days	β casein coated-gold nanoparticles (βCas AuNPs) 3 ng Au per 4.5 ng βCas 3–5 days (at different time intervals)	Inhibits Aβ plaque formation and reactive oxygen species formation. Recovering synaptophysin.	Locomotor activity	Javed et al., 2019
	Adult and larvae (5 dpf)	LDC8 (pyrazolotriazine derivative) Single dose	Decreases phosphorylated tau formation and inhibits GSK3β and CDK-5 activity	–	Reinhardt et al., 2019
Pentylentetrazole	Larvae 7 dpf	6-gingerol 12.5–37.5 μM 24 h	It acts as an inhibitor of NMDA containing NR2B channel	Locomotor activity	Gawel et al., 2021

*AChE, acetylcholine esterase; TNF α, tumor necrosis factor α; IL-1β, Interleukin-1β; PP2A, protein phosphatase 2A; GSK3β, glycogen synthase kinase 3β; cAMP, cyclic adenosine monophosphate; CDK-5, cyclin dependant kinase-5; NMDA, N-methyl D-aspartate.*

### Models for Adult Zebrafish

#### Y-Maze Test

Over the last couple of decades, there has been a significant rise in interest in the behavioral features of zebrafish. However, the current protocols are based on reinforcement/reward or avoidance and have long training periods. The advantages of Y-maze are that, it is simple and has quick training sessions, allowing specific training of memory as it does not include conditioned learning. It also minimizes factors that might influence performance such as emotional and motivational states. This task assesses the natural tendency of the fish to explore a novel arm when the mnemonic demand is less. As the retention time does last for more than a couple of hours, the performance of the fish can be evaluated multiple times (Benvenutti et al., 2021). Advanced free movement pattern (FMP) Y-maze assay is a combination of existing aspects of the Y-maze assay with certain modifications in the analysis. In a study conducted by Cleal et al., the effects of periodic doses of D-Amphetamine and nicotine on cognitive behavior in zebrafish in the FMP-Y maze were observed for 14 days. The observed tetragram based on the alteration patterns were analyzed. It was observed that there was an improved memory function in the zebrafish in initial stages after a short withdrawal period along with a decrease in cognitive flexibility which was observed on D-amphetamine administration but not when treated with nicotine (Cleal et al., 2021).

The apparatus used for this test is a three-armed glass tank whose length, width, and height are 25, 8, and 15 cm, respectively. Each arm can be associated with different visual cues and it should be ensured that zebrafish is not biased toward a particular clue (Cognato et al., 2012). Two arms of the maze are opened during initial training and one arm remains blocked. It is observed that the novel arm once opened for exploration, zebrafish normally tend to spend more time in this arm (Zanandrea et al., 2018). This indicates that they are recognizing the novel arm and are willing to explore it. For disease-induced (*via* inducers such as scopolamine) zebrafishes, it has been observed that they have a lower intention of exploration and tend to float around in a particular arm. To create an unbias environment with regards to the cues the maze is rotated for each experiment (Valu et al., 2021b).

Spontaneous alterations analysis allows the measurement of spatial memory of the animals. The following is used to calculate the same:


%⁢Alternation=Number⁢of⁢Alternations/[Total⁢number⁢of⁢arm⁢entries-2]×100

The higher the percentage of alteration the higher is the tendency to explore the novel arm which leads to an inference of low anxiety and good spatial memory (Kraeuter et al., 2019).

#### Hole Board Test

Hole board is a method normally used for the screening of prospective anxiolytic drugs. It is based on the hypothesis that the anxiety state of an animal is inversely proportional to its intention to look for baited holes. Initially, the animals are allowed to explore the experimental tank for 15 mins without any external visual cues in the open field and baited holes in the hole board. After habituation for a period of 4 days, training of zebrafish is commenced where only one hole of the whole board is baited. The time required to find the hole with the bait is noted and to avoid fixed directional swimming the fish is released into the tank from various locations. The maximum time given to each fish to find the baited hole is 3 mins (van der Staay et al., 2012; Bailey et al., 2015; Ruhl et al., 2015). This experiment tests the spatial cognition of the fish and therefore is an important model for testing of AD novel drug therapies.

#### T-Maze Test

T-maze is a method of assessing spatial memory, associative memory and learning in rodents and fishes. It has also been used in the evaluation of the pharmacological results of drugs in animal behavioral models. In zebrafish, this model is based on how quickly it can grasp a certain behavior through discrimination training which contains multiple sessions of training and contains a stimulus or food reward. The fish is trained to choose an arm through pavlovian conditioning (Gould, 2011; Bault et al., 2015).

The apparatus has one long arm which is the starting zone for the zebrafish and two short arms among which one is the favorable zone and the other one is the unfavorable zone These zones can also be color-coded. During training, the zebrafish is allowed to acclimatize within the two arms it can choose. If it chooses the wrong arm, it meets unfavorable conditions such as disturbance. Upon choosing the correct arm the zebrafish does not face any disturbance or can get a positive reinforcement such as food. On the day of acquisition, time spent in unfavorable and favorable zones is noted down. The control zebrafish will spend more time in the favorable zone and the AD model will not be able to develop such a bias. In the recent research conducted by Moreira et al., the effects of oxybenzone on behavior and cognition was tested by using various behavioral models including T-maze test. The results showed a decrease in the explorative instincts of the animal and impairment of the animal’s memory (Moreira and Luchiari, 2022).

#### Inhibitory Avoidance Paradigm

The apparatus consists of an aquarium which is of the dimensions 60 cm × 30 cm × 30 cm and contains about 10 cm water level. This aquarium is to be divided into two compartments with the help of a manually operating sliding door and the surfaces of each compartment are white and black, respectively. The black compartment is wired to a power source of 1-9V (AC). After allowing the fishes to acclimatize in both the compartments, an electric shock is given in the dark compartment for about 5 s and then the fish is returned to its home tank (Manuel et al., 2014). The following day the trained fish’s ability to avoid the dark compartment is observed. The fishes that do not enter the dark compartment in 3 mins are marked as successful in exercising their memory to avoid making the adverse choice and in the AD model, the fishes would show no such bias due to memory loss (Manuel et al., 2014).

#### Novel Tank Diving Test

For several animals, including zebrafish, new surroundings can be anxiogenic. This test has been considerably used in the modeling of anxiety and assessing the effects of anxiolytics. As this test is based on the idea of a novel surrounding, it is equivalent to the open field test generally performed in rodents. In this test, the initial response of an adult zebrafish is to stay at the bottom of the tank until they get accustomed to the rest of the tank (Stewart et al., 2012; Haghani et al., 2019).

In this method, the fishes are paired and transferred into a behavioral examination tank. After holding the fishes in the behavioral tanks, they are transferred into separate 100 ml beakers containing 25–30 mL water for immediate transportation into observational tanks. After the transfer, the fishes are observed in the new environment. The ability of the fishes to explore the novel tank is investigated. The more amount of time a fish spends at the bottom of the tank indicates high anxiety levels, which is a common manifestation in neurodegenerative disease models (Haghani et al., 2019).

Anwer et al. (2021) have recently hypothesized that this test when conducted in taller tanks yields a high repeatability.

#### Light Dark Test

The light-dark test is commonly used in the quantification of anxiety in rodents but in recent years has been also used for screening adult zebrafish. This model uses the nature of phototaxis displayed in fishes to evaluate the anxiety (Facciol et al., 2017). It is a straightforward and efficient method which does not require pre-training and enables understanding of pharmacological modifications in zebrafish. Further pharmacological studies observed that the method is sensitive to anxiolytic but not to the panicolytic drugs. In nature, a preference for the dark allows them to evade predators rather than being exposed in a white background. Anxiety inducing drugs alter zebrafish’s tendency to explore the tank and they stay in the dark region of the tank for longer periods compared to control, displaying negligible or low explorative preferences. The light-dark apparatus that is used for rodents is modified accordingly for application in zebrafishes. The tank has dimensions of 15 cm height × 30 cm length × 16 cm width (Stewart et al., 2011). The major differences in the apparatus are that it is sealed to avoid leakage of water which is filled to a height of 12 cm, there is also no sliding door between the two sides (light and dark) hence the fish are free to swim in both the compartments (Stewart et al., 2011). Currently several inducers of neurological diseases are being investigated to test for manifestations of symptoms like anxiety using this model. Decynium-22 was tested by Maximino et al. for inducing anxiety using the light dark test model (Maximino, 2021).

### Models for Larvae Zebrafish

#### Startle Response

Zebrafish larvae when exposed to abrupt stimuli of unexpected touch (tactile), loud sound (acoustic), or sudden bright light (visual) tend to react by a swimming burst to escape from the threatening stimuli (Fetcho and McLean, 2009). Startle response is important to understand whether the larvae have proper sensory and motor stimuli which are critical for survival (Troconis et al., 2017; Faria et al., 2019). The different times for development of different responses are listed in Table 4.

**TABLE 4:** Type of different startle responses and their time of development.

Type of startle response	time for development (days post-fertilization)	References
Tactile	2	Colwill and Creton, 2011
Visual	3	Ganzen et al., 2017; Tucker Edmister et al., 2022
Acoustic	5	Best et al., 2007

This behavioral model is important for neuropharmacological research to observe if the drugs being administered can restore the normal response to startling stimulus in larvae (Basnet et al., 2019; Banono and Esguerra, 2020). Locomotion analysis in larvae *via* high-throughput methods will be able to provide observations required for testing and identification of neuroactive compounds. Recent research by Png et al. (2021) used acoustic startle response with the help of 36 continuous mechanical taps, to observe the impairment of cognitive ability in response to 0.8 uM morphine administration for 5 days starting from 5 h post fertilization.

#### Optokinetic Response

The optokinetic response is first observed in zebrafish 73 h post-fertilization and it slowly develops until 4 days post fertilization where it reaches to 9/10th of an adult goldfish (Easter and Nicola, 1997). Around the time zebrafish larvae start hunting for food, the response is completely developed. There are various types of optokinetic responses but the horizontal optokinetic response is the only one studied in zebrafish and it is also commonly studied in other species. This response signifies a stereotyped eye movement in response to any form of movement within the field of vision (Huang and Neuhauss, 2008). To evoke an optokinetic response in zebrafish larvae an LCD screen displaying moving graphics or a rotating drum containing black and white stripes is used (Cameron et al., 2013).

Effect on the optokinetic response *via* chemicals that affect the central nervous system allows the study of Alzheimer’s induction and treatment by considering this response as an analysis parameter (Barbereau et al., 2021). This behavioral model has been used to study the effect of drugs that reduce anxiety such as fluoxetine. It was observed that the anxiolytic drug expunged the consequences (Hodges, 2021).

#### Thigmotaxis

When exposed to a new environment animals tend to have a natural tendency of moving to the periphery of the novel environment. This behavior, also known as the wall-hugging behavior, is used to estimate anxiety and it is conserved across all species (Simon et al., 1994; Colwill and Creton, 2011). Zebrafish larva shows thigmotaxis in response to novel environments at 5 days post fertilization. Exposure to sudden darkness or sudden light can elucidate this behavior in zebrafish (Liu et al., 2016). The test should be performed in an arena where there is enough space to distinguish between inner and outer zones so the choice is usually a 24 well plate (Schnörr et al., 2012).

This test can be used to study anxiety-like behavior in the zebrafish (Cho et al., 2012). Thus, using this behavioral model one can study the induction of Alzheimer’s like phenotype and efficacy of anxiolytic drugs (Ahmad and Richardson, 2013). Recent studies have been conducted to test the anxiolytic effect of compounds like Buspirone hydrochloride by observing thigmotaxic behavior in zebrafish as it was a responsive and straightforward way for evaluating the efficacy of similar anxiety reducing drugs (Abozaid and Gerlai, 2022).

#### Optomotor Response

Zebrafish can be exposed to aversive and non-aversive visual cues on an LCD screen monitor. For responsive cues, 15 zebrafish larvae are placed in a single petri dish and then placed on a monitor. The zebrafish is exposed to moving red and white stripes 5 days post-fertilization (Fleisch and Neuhauss, 2006; Creton, 2009; Nery et al., 2017). The directions of the stripes are alternated every 1 min with a 5-s interval, where the cue is faded (Karaduman et al., 2021). The animals usually tend to swim in a direction similar to the movement of the stripes. The petri plate is divided into three zones to observe the number of fishes present in the correct zone. In aversive cues, the LCD monitor displays a red bouncing ball. The animals will try to swim to the non-stimulated area of the petri dish to escape the bouncing ball (Pelkowski et al., 2011; Nery et al., 2014). These tests help in determining the cognitive performance of animals (Karaduman et al., 2021). Quinpirole, a compound that induced anxiety-like symptoms by targeting dopaminergic signaling, was observed to impair optomotor response (Nabinger et al., 2021).

#### Prey Capture

After hatching, zebrafish embryos start swimming toward the prey, a behavior that is essential for survival (Muto and Kawakami, 2013). To test this behavior a small air bubble released in the test arena is utilized as prey (Gahtan et al., 2005). In the assay, the latent period before the attack on the prey, the number of attacked or captured prey, and the efficiency with which the prey is captured are the parameters measured (Tierney, 2011). The convergence of eye movement marks the onset of hunting behavior and the prey attack is defined by the biting motion of larvae (Bianco et al., 2011). This behavior involves decision making therefore can be used to assess cognitive behavior (Formella et al., 2018).

## Conclusion and Future Prospects

The zebrafish model is a robust system for the physiological and genetic study of AD (Xia, 2010). The advantages of the zebrafish models are that they allow sizable forward genetic screening, investigation of different properties such as temporal, spatial, and also real-time observation of the pathological changes *in vivo*, exploration of behavioral and pharmaceutical in both larvae and adults (Lorent et al., 2004). Over the years, extensive research has provided abundant evidence and essential information about the correlation between disease and its development in zebrafish (Brun et al., 2021). Due to advancements in modern technology, the zebrafish system can be established as a substitute to mammalian models in drug development, target identification, and validation along with providing shorter routes for novel therapeutic strategies (Chakraborty et al., 2009). The larva-adult duality in the zebrafish model has a large-scale utilization for preclinical studies before rodent models for validating novel therapies (Bailey et al., 2015). The article sheds light on the use of behavioral models of both the larva and adult zebrafish along with the different neurotransmitter pathways that regulate them but strategies to overcome the complexities are yet to be established (Santana et al., 2012; Saleem and Kannan, 2018). Zebrafish as a model could assist in further understanding neuroanatomical circuits and their role in neurodegenerative disorders (Stewart et al., 2015). Further research in regard to zebrafish telencephalon is required to gain an understanding of the neurological and molecular mechanism at the onset of AD. This study would allow us to extrapolate the observations and corelate them to the early biomarkers of mammalian AD commencement in the hippocampus. This would assist in the progress of novel preventive therapies. It can be concluded that the zebrafish is a promising model for gaining a perception of mechanisms of AD but requires further optimization to be indispensable to novel drug screening.

## Author Contributions

GK and AU visualized the presented idea, contributed to the manuscript writing, supervised the project and corrected, revised, and approved the manuscript. MB, SB-P, and AS contributed equally to doing literature searches and in the preparation of the manuscript. All authors contributed to the article and approved the submitted version.

## Conflict of Interest

The authors declare that the research was conducted in the absence of any commercial or financial relationships that could be construed as a potential conflict of interest.

## Publisher’s Note

All claims expressed in this article are solely those of the authors and do not necessarily represent those of their affiliated organizations, or those of the publisher, the editors and the reviewers. Any product that may be evaluated in this article, or claim that may be made by its manufacturer, is not guaranteed or endorsed by the publisher.
